# Closing a Large Maxillary Median Diastema using Bapat Power Arm

**DOI:** 10.5005/jp-journals-10005-1435

**Published:** 2017-06-01

**Authors:** Shirish M Bapat, Chanchal Singh, Prashant Bandejiya

**Affiliations:** 1 Director, Principal, Professor and Head, Department of Orthodontics and Dentofacial Orthopedics K.D. Dental College & Hospital, Mathura, Uttar Pradesh, India; 2 Professor and Head, Department of Pedodontics and Preventive Dentistry, K.D. Dental College & Hospital, Mathura, Uttar Pradesh, India; 3 Postgraduate Student, Department of Orthodontics and Dentofacial Orthopedics K.D. Dental College & Hospital, Mathura, Uttar Pradesh, India

**Keywords:** Bapat power arm, Bodily movement, Diastema closure.

## Abstract

**Aim:**

The aim of this study is to present a case of large maxillary median diastema closed by bodily movement of central incisors using Bapat power arm (BPA).

**Materials and methods:**

After extraction of mesiodens, a power chain with a force of 120 gm was applied to BPA ligated to preadjusted edgewise brackets bonded to maxillary central incisors to move them over round steel wire for closure of resultant diastema. Bonded retainer was placed after the closure of median diastema.

**Results:**

The median diastema was completely closed in 5 months period with almost bodily movement of incisors, which was confirmed by periapical X-ray.

**Conclusion:**

Bapat power arm was efficient in closing diastema without any discomfort or injury and was well accepted by the patient.

**How to cite this article:**

Bapat SM, Singh C, Bandejiya P. Closing a Large Maxillary Median Diastema using Bapat Power Arm. Int J Clin Pediatr Dent 2017;10(2):201-204.

## INTRODUCTION

The median diastema is a condition of having central incisors with intervening space between them. Such a space, called diastema, is certainly annoying for affected individuals as it is easily noticed and esthetically displeasing. Therefore, it is a matter of great concern for the child as well as parents.^1^ Such spaces are usually seen in early mixed dentition period, around 6 to 9 years, but spontaneously disappear by the time maxillary permanent canines erupt and often require no intervention.^2-4^ However, in some children, diastema continues to persist till adult age.

Occurrence of such diastemas is attributed to mul-tifactorial etiological reasons like normal physiological event, genetic and racial predisposition, developmental defects and congenital anomalies, local physical impediments, muscular imbalance, pernicious habits, dental anomalies, or iatrogenic result of orthodontic procedures like rapid expansion.^5,6^ Prevalence of median diastema is high around age 6 to 7 years (40-50%) but diminishes by 15 years (5-7%).^7,8^ It is reported more in females at the age of 6 years but more males present with diastemas by 14 years of age than females.^6^ Diastemas of less than 2 mm in 9-year-old children generally close spontaneously.^6,9,10^

## AIM

The aim of this study is to present a case of a large median diastema closed by bodily movement of maxillary central incisors using Bapat power arm (BPA).^11^

## CASE REPORT

**Figs 1A and B:**
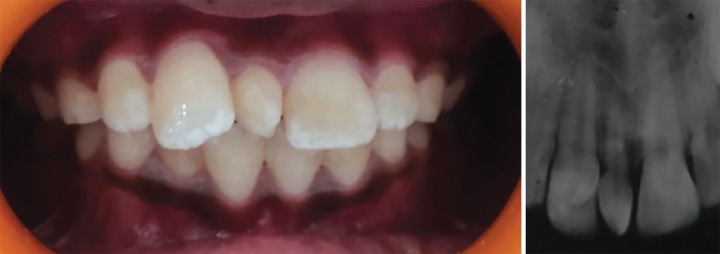
Pretreatment view showing mesiodens: (A) Teeth front view; and (B) IOPA radiograph

A 9-year-old male child was brought to the Department of Paediatric Dentistry of K.D. Dental College and Hospital, Mathura, Uttar Pradesh, India, by his parents with a complaint of an ugly looking small tooth between maxillary permanent central incisors (Fig. 1). Intraoral examination and intraoral periapical (IOPA) radiograph confirmed the maxillary mesiodens (Fig. 1). The posterior teeth had unsettled occlusion because of premature occlusal contacts in incisor region. After taking all necessary records and informed consent, the mesiodens was extracted under local anesthesia (2% lignocaine hydrochloride) at the Paediatric Dentistry Department. Patient was called back after a week. When he returned, an 8 mm large diastema was clearly visible while speaking or smiling, which further added to the patient’s psychological trauma. The parents stated that the child had developed low self-esteem and depression and could not concentrate in his studies because of a large space between his front teeth. It had affected his confidence.

**Figs 2A and B:**
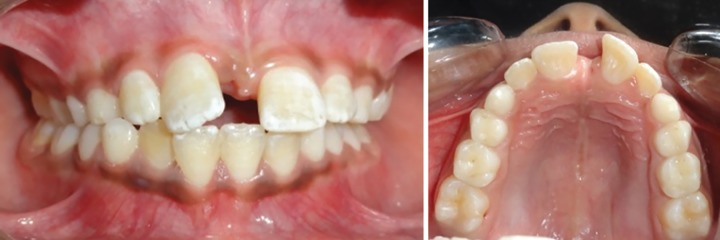
Pretreatment view after extraction of maxillary mesiodens: (A) Intraoral frontal view; and (B) intraoral occlusal view

Orthodontist’s opinion was sought. The orthopan-tomogram (OPG) was taken, which revealed that the permanent canines were unerupted and were erupting in mesial angulation and the premolars were present in their normal position beneath the roots of deciduous molars (Fig. 2). The incisor occlusal interference caused cusp to cusp posterior occlusion.

**Fig. 3:**
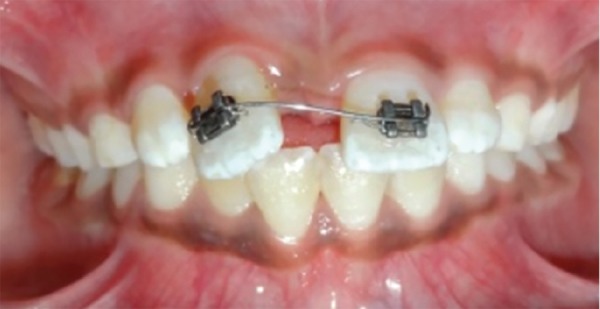
Initial sectional wire

**Fig. 4:**
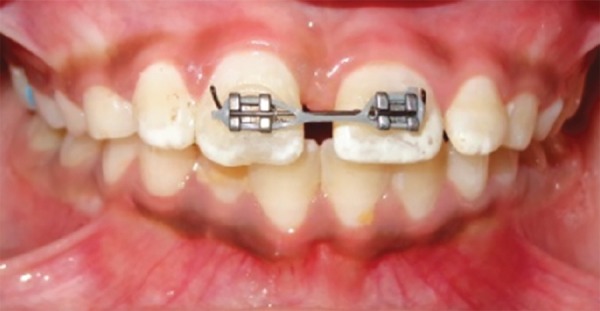
Initial correction done with sectional wire

**Fig. 5:**
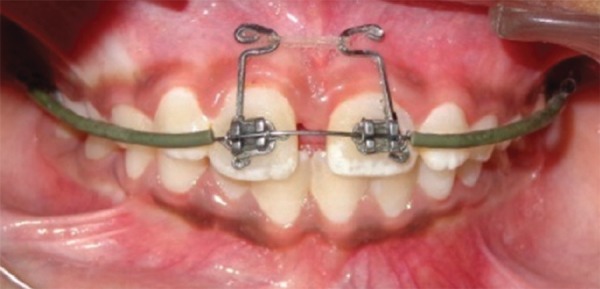
Diastema closure started with sliding movement of central incisors along full arch wire with curve of Spee with power chain applied to BPA

It was decided to close this large diastema of 8 mm by bodily movement of central incisors. Edgewise brackets (0.022"" × 0.028"" slot) were bonded on the maxillary central incisors and a 0.018"" round nickel-titanium sectional wire was initially ligated for their derotation and alignment (Figs 3 and 4). Once that was accomplished, a 0.018"" round stainless steel full-arch wire with curve of Spee was ligated for slight intrusion of central incisors. A rubber sleeve was placed on the large posterior sections of wire to prevent trauma to cheek. Thereafter, closure of median diastema was started using power chain with a force of 120 gm applied to BPA^10^ ligated to both central incisors (Fig. 5).

**Fig. 6:**
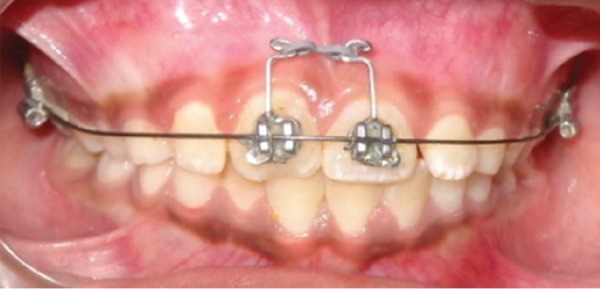
Median diastema closed and spaces opened mesial and distal to lateral incisors. These residual spaces mimic physiological spaces which will help in eruption of permanent canines

**Fig. 7:**
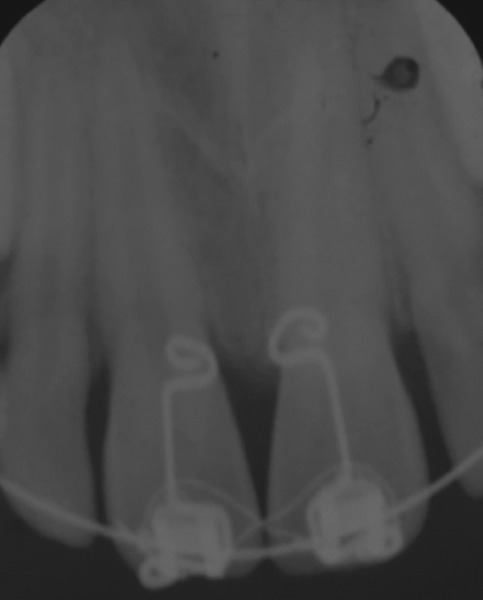
Posttreatment IOPA radiograph showing almost parallel roots of central incisors

**Fig. 8:**
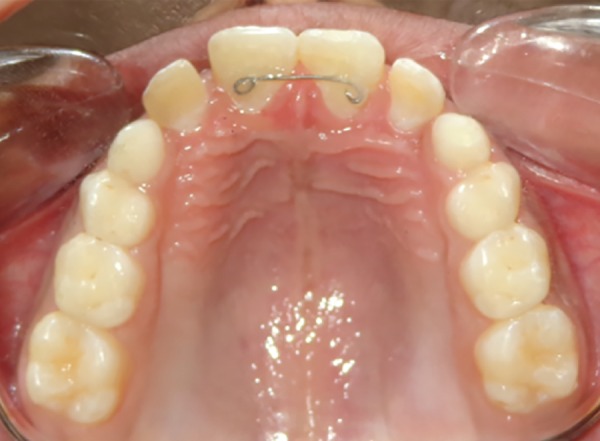
Bonded palatal retainer was placed after diastema closure

**Fig. 9:**
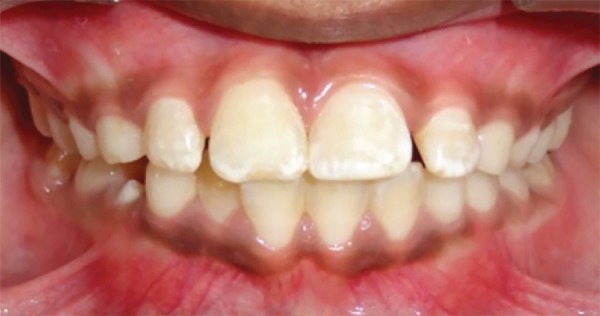
Posttreatment result

The large median diastema was completely closed in 5 months period. Spaces opened distal to central incisors and distal to lateral incisors and posterior occlusion settled (Fig. 6). The brackets were then ligated by figure of 8 wire ligatures to maintain diastema closure. Intraoral periapical radiograph was taken, which revealed that the central incisor roots were almost parallel (Fig. 7), indicating a bodily movement of these teeth during dia-stema closure. Thereafter, direct bonded flexible spiral wire retainer was placed on palatal surfaces of crowns of central incisors well clear of incisor occlusion (Fig. 8). The brackets, wire, and BPA were then removed and photographs, IOPA and OPG radiographs, and study casts were obtained. The final result was esthetically pleasing (Fig. 9), and hence, psychologically satisfying for the patient.

## DISCUSSION

Diastemas are major esthetic concerns^1,12^ and hence, are an important reason for seeking orthodontic treatment for their closure by many patients.^6^ The management of midline diastema depends upon their etiology.^13^ Habit-breaking appliances are effective in treating diastemas attributed to tongue thrust or finger-sucking habits. Small diastemas can be closed by using removable appliance with finger springs.^5,6^ Wider diastema needs closure by fixed appliance for correcting and controlling crown and root angulations and maintaining overbite control.^5,6,9^ Prosthetic replacement of missing teeth and other restorative procedures like veneers, crowning, and composite buildups are commonly used in patients with tooth size discrepancies or when such needs are necessary in other patients. These treatment modalities should be deferred till eruption of permanent canines.^6^ Surgical procedures like frenectomy, interdental corticotomy, and glossec-tomy have been proposed to treat high, enlarged labial frenal attachments, large interdental alveolar septum or interdental alveolar cleft, and large tongue respectively.^6^

Removable appliances generally close diastemas by tipping the crowns of incisors. They do not provide effective vertical or torque control, which results in a strong tendency toward relapse.^6^ Fixed appliances can move teeth bodily. Tipping can be minimized by giving a υ-bend in the center of the wire in vertical plane. Double helical loop (e.g., M spring) can reduce mesial crown tipping during diastema closure.^5,6^ However, a 2x4 appliance or utility arch can provide better vertical and torque control of incisors during closure of midline diastema and can also retract incisors.^6^ A gentle curve of Spee should be incorporated in the plain arch wire for intrusion of extruded incisors. As diastemas are commonly found in pediatric patients, a good interdisciplinary approach by pedodontist and orthodontist is beneficial for the patient for their management.

The median diastemas have a strong tendency to recur after their closure.^4^ Hence, a lingually bonded fixed retainer is recommended.^6^ Edwards^4^ found diastema relapse in 84% of his sample with a strong correlation between labial frenum and diastema relapse. Another study found midline diastema recurrence in 60% of the sample with stronger correlation of relapse with larger initial diastema width, relapse of overjet, and intermaxillary osseous cleft and concluded that midline diastema closure is highly unstable, hence, needs lifetime wear of maxillary fixed retainer.^14^ Inadequate root parallelism at the end of treatment has been also cited as a reason for median diastema relapse.^4,15^ However, some mesial inclination of central incisors is preferred.^14^ One follow-up study reported relapse of median diastema in 49% patients and found wider initial width of diastema and fremitus of maxillary incisors strongly associated with space opening.^16^ Though the prevalence of mesiodens is reported to be only 0.15 to 2.2% of the population with a preference to males,^17^ their presence in the maxillary arch could be deleterious from the esthetic and functional points of view.^18^ Hence, these patients need prompt treatment.

Bapat power arm was developed to apply a force more apically, about 4 mm above cementoenamel junction, closer to center of resistance of the tooth to enable more bodily movement by sliding mechanics with tipping and rotational control of crowns. Large diastema closure with force applied at the bracket level leads to mesial tipping of crowns and distal flaring of roots. Teeth tipped in such a manner remain unstable. This results in diastema recurrence by relapse over a period of time. Hence, the force was applied to BPA rather than to the brackets. The central incisors moved bodily. This was indicated by the fact that the axial inclination between the two central incisors was maintained constant, i.e., 7°, from start to end of space closure. There was no discomfort, nor any injury to lip or gingiva. Patient adapted well to BPA. Bapat power arm is simple in design and easy to use as it obviates the need of bonding or banding of the tooth, which is required for other power arms. It is prefabricated, hence, saves chairside time too.

## CONCLUSION

Large median diastemas cause psychological concerns among children and their parents and require closure by bodily movement of central incisors. Such a large median diastema was successfully closed by bodily movement of maxillary central incisors using BPA.
